# Was the “ABC” Approach (Abstinence, Being Faithful, Using Condoms) Responsible for Uganda's Decline in HIV?

**DOI:** 10.1371/journal.pmed.0030379

**Published:** 2006-09-12

**Authors:** Elaine M Murphy, Margaret E Greene, Alexandra Mihailovic, Peter Olupot-Olupot

## Abstract

Background to the debate: Uganda is one of the few African countries where rates of HIV infection have fallen, from about 15 percent in the early 1990s to about five percent in 2001. At the end of 2005, UNAIDS estimated that 6.7 percent of adults were infected with the virus. The reasons behind Uganda's success have been intensely studied in the hope that other countries can emulate the strategies that worked. Some researchers credit the success to the Ugandan government's promotion of “ABC behaviors”—particularly abstinence and fidelity. Uganda receives funds from the United States President's Emergency Plan for AIDS Relief, which promotes the ABC approach with a focus on abstinence-driven public health campaigns. Other researchers question whether the ABC approach was really responsible for the decline in HIV infection. Critics of the ABC approach also argue that by emphasizing abstinence over condom use, the approach leaves women at risk of infection, because in many parts of the world women are not empowered to insist on abstinence or fidelity.

## Abstract 2

Murphy and Greene say policies to advance women's status were crucial to the success of "the ABCs" in reducing HIV rates in Uganda. But Mihailovic and Olupot-Olupot argue that by emphasizing abstinence, the ABCs are political and not evidence based.

## Elaine Murphy and Margaret Greene's Viewpoint: Policies to Advance Women's Status Were Crucial to the ABCs' Success in Uganda

A debate continues to simmer over the much-publicized “ABC” approach to HIV/AIDS prevention, most narrowly defined as: Abstain, Be faithful or reduce the number of your sex partners, and/or use a Condom. The discussion has become polarized in part because for some, the ABCs are synonymous with the promotion of abstinence-only sex education programs for youth, an area of considerable controversy [1] that seems to pit political and religious conservatives against their liberal counterparts.

In addition, although ABC behaviors have been credited with Uganda's dramatic decline in HIV rates [2–5], questions remain as to whether the ABC-related behavior changes are attainable in other developing countries, given many women's relatively limited control over their sexual relationships. Influential AIDS policy makers have expressed doubt that ABC-related behavior changes can take place in settings where women seem to have little control over their sex lives. On the eve of the 2004 International AIDS Conference in Bangkok, for example, the deputy executive director of UNAIDS observed that, “Most of the women and girls, as much in Asia as in Africa, don't have the option to abstain when they want to. Women who are victims of violence are in no position to negotiate anything, never mind faithfulness and condom use” [6]. An influential woman's advocate reinforces this view: “Most prevention messages…focus on the ‘ABC’ approach to fighting HIV-AIDS…. While important messages, these things are often not within women's power to control” [7].

### Gender Inequity and the Spread of HIV

These concerns are valid. Gender inequity is an indirect but powerful factor in the sexual spread of HIV. Gender norms create inequality between the sexes in power, autonomy, and well-being, typically to the disadvantage of females [8]. An extensive literature on women's subordinate status in most societies—but particularly in poor countries—points to widespread patterns of male privilege, visible in social discrimination such as lower levels of investment in the health, nutrition, and education of girls and women [9–12]. Institutionalized economic inequalities keep land, money, and other resources out of women's hands, making women financially dependent on men, less likely to be able to negotiate sex with a partner, more likely to practice survival or transactional sex, and more subject to violence [13,14]. Violence against women varies by country but is a global problem and a well-documented risk factor for HIV [15,16]. In many countries, women's sexual subordination exposes them to elevated reproductive health risks: coerced sex and rape, maternal mortality, unsafe abortion, and sexually transmitted infections (STIs), including HIV [17].

However, ABC behaviors were attainable in Uganda, a society where many women had little power at the outset of the AIDS epidemic. Fortunately, a “this could not work here” attitude did not deter Uganda from moving forward to implement its wide-ranging HIV prevention program and adding gender-related elements when it became clear that this strategy was necessary. Other countries show signs of desirable change as well. In Zambia and Kenya, ABC-related behavioral changes are emerging among youth and adults, accompanied by reduced rates of incidence [2,18,19]. Thailand, likewise, was not only successful in promoting—and requiring—condom use in brothels, but also in bringing about changes in fidelity and partner reduction among the general population—particularly young men—through community mobilization [20]. Research in Malawi provides additional evidence that poor women's protective strategies in response to the threat of AIDS have been overlooked by many AIDS prevention programs [21].

### ABCs in Uganda: Outcomes, Not Strategies

ABC-related behavior changes have taken place in Uganda and a small number of other countries not only because fear of AIDS has led to protective action by men and women but because many interventions have also directly addressed gender inequities. Greater openness about the dangers of unprotected sex and challenges to women's subordinate role in sexual decision-making have helped to create an environment in which many more women have found it easier to abstain, reduce their number of partners, and/or negotiate condom use [22].

One important point is that abstaining from sex, being faithful, and using condoms—ABC-related behaviors—are outcomes of prevention strategies, not strategies in themselves. The reasons the ABC messages were exceptionally successful in Uganda extend beyond the content of the messages themselves. Abstinence, being faithful, or reducing one's number of partners were indeed promoted among the general public, and condoms were emphasized for high-risk groups. But Uganda's success in bringing about behavior change relied primarily on extensive social mobilization at every level and strong political leadership from its president, Yoweri Museveni, who particularly emphasized fidelity [23].

### Challenging Gender Norms Supported ABC Behaviors in Uganda

The ABC behavior changes that cut Uganda's HIV prevalence by about two-thirds were the outcome of a massive, nationwide social mobilization against AIDS [2,4,23]. The messages were not merely moral exhortations by religious leaders, although religious bodies along with schools and many other civil society groups were actively involved. Many feel that President Museveni's leadership was instrumental in bringing about widespread changes in sexual attitudes and practices.

In 1986, Museveni, a hero of Uganda's civil war, declared that the nation was still at war and the enemy was AIDS. He undertook public education on HIV, and his ongoing series of radio AIDS messages urged men in particular to change their behavior—to be sexually responsible—and encouraged “mutual respect” between spouses, widely interpreted as mutual fidelity. Women's groups also played a key role by mobilizing and publicizing women's difficulty in controlling the circumstances under which they had sex. Museveni responded by highlighting the importance of promoting sexual behavior change and equity between men and women (F. Kitabire, personal communication). In a 2001 keynote address to the organization that sponsors the Africa Prize for Leadership, a prize the country of Uganda and President Museveni had won in 1998, Museveni spoke out on these issues:

Permit me to tell you the obvious. In the fight against HIV/ AIDS, women must be brought on board. In sub-Saharan Africa, most women have not yet been empowered and men dominate sexual relations. To fight this epidemic, the women must be empowered to take decisions about their sexual lives, and women in Uganda have been empowered and participate today at all levels of governance. This has made them more assertive of their rights than ever before. To fight AIDS effectively, we must empower women [24].

President Museveni ensured that affirmative action policies that enabled women to participate in local and national politics were written into Uganda's national constitution. Museveni also created a Ministry of Women's Affairs, charged with vigorous enforcement of laws against sex with minors. Both public and private school systems designed and implemented sexuality education, which included gender equity messages. The Museveni government developed both macro- and micro-credit schemes for women and fostered government and nongovernmental programs that promoted gender equity among women, men, and youth. President Museveni went so far as to propose a law—unfortunately, unsuccessful—against mate rape to the Parliament [25]. Though divorce laws favor men and it is still difficult for women to divorce or renounce abusive husbands, organizations such as the Uganda Association of Women Lawyers have opened the door for abused women to do so [26,27]. These actions are likely to have contributed to changing gender dynamics.

### ABCs in Uganda—Measuring the Impact

HIV prevalence in Uganda peaked in 1991 at about 15 percent of the adult population and declined to about five percent in 2001 [28]. Trend data reveal epidemiologically significant behavior changes in Uganda, especially in reduced numbers of sexual partners and later sexual debut. Concurrent partner reduction among both men and women was a key factor in the reduction of HIV infection in Uganda [4,29]. Significantly, much of the most substantial behavior change occurred among men [30].

According to Demographic and Health Surveys (DHS) and other research, median age at first sex rose by 1.2 years for girls and 1.7 years for boys between 1989 and 2000 [31]. The percent of 15–19-year-old women ever having sex dropped from 74 percent to 51 percent; among men of the same age, the figure dropped from 68 percent to 42 percent. The percentage of Ugandan women aged 15–24 reporting premarital sex also declined from 53 percent to 16 percent; among young men, the decline in premarital sex was from 60 percent to 23 percent [28]. Data from Uganda's 2000–2001 DHS show a remarkably high 78 percent of unmarried 15–19-year-old men and women reporting zero sexual partners in the past year [31].

Between 1989 and 1995, the WHO Global Programme on AIDS and UNAIDS reported that the percent of Ugandan women with one or more casual partners dropped from 16 percent to six percent, while the same figures for men went from 35 percent to 15 percent [28]. Perhaps even more remarkably, the number of men reporting three or more partners declined from 15 percent in 1989 to three percent in 1995 [18]. Reported extramarital sex among women in Uganda is now very rare at one to three percent [18]. Anecdotal evidence from field researchers suggests that among younger men, having an STI, once considered a badge of manhood, is now in the era of AIDS considered a matter of shame or stupidity (S. Watkins, D. Halperin, personal communications). The “B” message may also have been relevant for some women, especially younger unmarried women who were sexually active and had multiple partners [2].

Uganda was relatively slow in promoting condoms. For the first few years after ABC messages were promulgated, the focus was on abstinence and partner reduction, A and B. Between 1988 and 1995, the percent of married Ugandan women who were currently using condoms rose from 0 percent to 0.8 percent and from 0 percent to 15.4 percent for sexually active unmarried women [31,32]. This trend continued between 1995 and 2000: the percent of married Ugandan women who were using condoms rose from 0.8 percent to 1.9 percent and for sexually active unmarried women it rose from 15.4 percent to 29 percent. Between 1995 and 2000 condom use among married men rose slightly from three percent to five percent. However, among unmarried men aged 15–24, reported condom use at last sex increased sharply—from 39 percent to 57 percent [32]. Thus marital use of condoms increased only slightly while non-marital increases were dramatic. However, a large proportion of sexually active unmarried youth, particularly young women, do not use condoms at all, and among those who do, there is no data on how correct and consistent their condom use is. More work is needed in this area to normalize condom-carrying by women.

Survey data also show a large proportion of women reporting that they can refuse unwanted sex under specific circumstances. Remarkably, in the 2000–2001 Uganda DHS, 91 percent of women said they could refuse sex with their husbands if they knew their husbands had STIs [31], a somewhat higher percentage than in several other African countries (73 percent in Malawi, 87 percent in Rwanda, 82 percent in Tanzania, and 71 percent in Zimbabwe) [33–36]. Even discounting some percentage points for social desirability factors, the levels in these countries are unexpectedly high. There is also evidence that some sex workers are taking effective steps to protect themselves [37]. While there are also large numbers of women who are sexually victimized, women in poor countries are not homogeneous in terms of their vulnerability or ability to protect themselves—and programs should be tailored accordingly.

### Where Do We Go from Here?

The importance of including gender-related interventions is a lesson to be learned from Uganda, where policies to advance women's status were part of the ABC strategy. In the context of Uganda's political leadership, nationwide social mobilization, and gender empowerment policies, both women and men benefited and HIV prevalence declined. However, in most developing countries, HIV prevention programs fail to address the pervasive challenges of gender inequity.

Uganda provides one model, albeit far from perfect, and there are other successful or promising efforts around the world that challenge gender norms. In many of these programs, male involvement plays a central role [38]. While there are many examples of separate programs for women and men, we must not forget the importance of working with partners together. Research shows that dealing with couples is often more effective than working with men or women individually in terms of family planning and HIV counseling [39]. There are, however, few organized programs for couples.

To be effective in the long term, programs must work to transform the gender norms that make women subordinate to men and encourage men to take risks in the name of masculinity. To achieve this goal, special efforts must be directed to men and women, separately and together, and to policy makers. There are encouraging signs that increasingly women have acted to protect themselves from HIV, and that men are questioning the dimensions of masculinity that harm their health, and we need to learn more about their stories. We must listen to women and men in order to address their needs; this in itself constitutes a worthwhile AIDS prevention research agenda. It makes no sense either to dismiss or to promote “the ABCs” as if this were a strategy or program rather than behavioral responses to social mobilization, leadership, and empowerment. We must work to create an environment that makes these behavioral responses logical and possible for both women and men. The evidence suggests that these efforts will reap rewards in declining HIV rates.

## Alexandra Mihailovic and Peter Olupot-Olupot's Viewpoint: An Emphasis on Abstinence Takes Away from Evidence-Based Prevention

With nearly 16,000 new infections daily, mostly occurring in sub-Saharan Africa, HIV/AIDS is the world's most urgent health problem. Public health efforts to reduce new infections and treat infected people are increasingly complex due to politicization of the epidemic and to public health interventions that reflect specific groups' religious values.

The largest source of international funding for HIV/ AIDS, the US President's Emergency Plan for AIDS Relief (PEPFAR), has increased its funding by 55 percent over the past two years with a focus on abstinence-driven public health campaigns [40]. One-third of the funds are to promote abstinence, with future funding conditional upon demonstrated activities [41].

Uganda is one of the 15 focus countries currently receiving PEPFAR funding. Recent political and religious influences on Uganda's response to the epidemic, including guidance from PEPFAR, have led to the country promoting the “ABC” campaign. But the success of abstinence-focused campaigns is bitterly disputed [42]. PEPFAR has referred to its focus on abstinence as an “evidence-based” risk-reduction strategy, citing failure rates for condoms [43]. Sadly, PEPFAR fails to address the failure rates with abstinence. In our view, there are several important shortcomings of the ABC campaign.

### Is Uganda Really an “ABC” Success Story?

Uganda's successes over the course of the HIV/AIDS epidemic must not be overlooked. It was the first sub-Saharan country to take an active role in acknowledging HIV/AIDS in the community and implementing interventions in the 1980s that successfully reduced prevalence rates in the 1990s. The Ugandan AIDS Commission developed a clear policy by 1986, focusing on mass education and awareness campaigns, blood system safety, voluntary counseling and testing, prevention of mother-to-child transmission, women's empowerment, and treatment [44]. However, abstinence was not a primary focus of the public health campaigns during the 1990s [45].

### Political Motivations

Uganda has a complex mix of citizens divided north to south by a 20-year civil conflict. The increased humanitarian aid for HIV/AIDS and strong statements of response in the south has taken the international eye away from the fragile political situation which has left 1.6 million people living in internally displaced person (IDP) camps [46]. The HIV/ AIDS prevalence rates in IDPs are thought to be similar to rates in people living in urban areas, and may be attributed to insufficient condom provision and inadequate sexual education in an area where control over sexual exposure to HIV is limited [47,48]. The success that has been achieved in delivering increased sexual education to those in the southern areas of the country should not allow us to ignore the complexity of the HIV/AIDS epidemic and the neglected humanitarian crisis still present in Uganda.

Politicians have criticized condom promotion as “pushing young people into sex” and have described pre-marital sex as “deviant and immoral.” Suggestions of a national “virgin census” on World AIDS Day in 2004 raised fears that children could be forced to submit to intrusive medical tests or breach of confidentiality [42]. Such extreme views about condoms and premarital sex have no place in rationally confronting a disease as complex and far-reaching as the HIV/AIDS epidemic in Africa. Political inclinations towards supporting one particular approach, without due consideration of local social, cultural, and biological factors, ignore the diverse political and demographic settings of the epidemic.

### The Problems with A and B without C

By focusing on individual behaviors, the ABC approach does not acknowledge the underlying factors that make people vulnerable to HIV/AIDS. The ABC strategies dismiss the real social, political, and economic causes of the epidemic, and end up blaming infected people, because it is implied that they failed to adopt and practice the ABCs. The ABC approach ignores vulnerable populations, such as sex workers and those who lack the ability to negotiate safe sex. It further fails to address non-heterosexual risk groups such as men who have sex with men and intravenous drug users.

PEPFAR's ABC guidance contains rules for country teams to follow in developing and implementing their sexual prevention strategies, including parameters on the prevention messages that may be delivered to youths. Specifically, although funds may be used to deliver age-appropriate AB information to in-school youths, ages 10–14 years, the funds may not be used to provide information on condoms to these youths or distribute condoms in any school setting, let alone youth out of school. And yet as many as 16 percent of all women in Uganda have sex before the age of 15 years [49].

The ABC campaign assumes abstinence will allow young women to focus on going to school, controlling their relationships, and becoming socially empowered, and yet it fails to acknowledge the social circumstances driving sex in the first place. Many sexual relationships include transactional or commercial sex, in order to pay for post-secondary schooling, to gain fi nancial independence from family obligations, or to provide adequate resources for those contained in IDP camps [50]. Encouraging abstinence, while at the same time excluding sexual education and protection against HIV, puts these girls at great danger of exploitation and ignorance, depriving them of the opportunity to learn the needed tools to approach sexuality in a healthy and informed manner.

Ironically, by promoting marriage (Be faithful) as a prevention measure, this campaign negates one of the highest risk groups in Africa: monogamous, married women [50]. Surveys suggest a high incidence of extramarital sexual activity and STIs among some married men [50]. It is still widely believed in Uganda that women have no right to deny their husbands sex [51]. The assumption of the campaign that sex is a rational act and that women have the autonomy to choose abstinence ignores the forces behind the initiation of sex. The presumption that marriage is somehow protective is misleading and potentially dangerous for young women already deprived of proper sexual education.

The enormous disservice done by the recent campaign to discourage condom use (due to the assumed link to promiscuity) cannot be overemphasized. The effectiveness of condom use for prevention of HIV/AIDS is the most likely explanation for Uganda's early successes [45]. Deemphasizing the importance of condom use has the serious potential to hurt local prevention efforts. A 2005 study by researchers at Makerere University and the AIDS Information Centre showed that Ugandans aged 19–25 years were more concerned about getting pregnant than becoming infected with HIV; when condoms were used, they were primarily considered contraceptive tools rather than protection against infections [52]. The confusion in young women and men who initially doubted the efficacy of condoms has only been amplified by these new efforts by the Ugandan government [49].

### Recommendations

We still don't know the most effective strategy for decreasing the number of new cases of HIV in Africa. Given the lack of evidence underpinning the abstinence strategy in the first place, it is crucial that condom use and education be emphasized if PEPFAR is to reach its target of preventing 7 million new infections by 2010 [53].

We need to ensure that the messages that we are sending to youth are not contradictory and that schoolteachers are adequately informed to provide objective counseling to sexually active pupils. We need to ensure that the special needs of vulnerable and oppressed populations are addressed. Urgent steps are required to provide factual and empowering information about each of the ABC components in order to counter misinformation, fear, and stigma. Finally, we need to increase and ensure free and widespread testing so that individuals can be empowered to protect themselves as well as their loved ones by being informed of their own and their partner's infection status. The sooner we confront HIV/AIDS as the multifaceted and complex issue it is, the sooner we can make important steps towards progress in HIV prevention.

## Abbreviations

DHS: Demographic and Health Surveys
IDP: internally displaced person
PEPFAR: President's Emergency Plan for AIDS Relief
STI: sexually transmitted infection
